# Early mortality among children and adults in antiretroviral therapy programs in Southwest Ethiopia, 2003–15

**DOI:** 10.1371/journal.pone.0198815

**Published:** 2018-06-18

**Authors:** Hailay Abrha Gesesew, Paul Ward, Kifle Woldemichael, Lillian Mwanri

**Affiliations:** 1 Public Health, Flinders University, Adelaide, Australia; 2 Epidemiology, Jimma University, Jimma, Ethiopia; Azienda Ospedaliera Universitaria di Perugia, ITALY

## Abstract

**Background:**

Several studies reported that the majority of deaths in HIV-infected people are documented in their early antiretroviral therapy (ART) follow-ups. Early mortality refers to death of people on ART for follow up period of below 24 months due to any cause. The current study assessed predictors of early HIV mortality in Southwest Ethiopia.

**Methods:**

We have conducted a retrospective analysis of 5299 patient records dating from June 2003- March 2015. To estimate survival time and compare the time to event among the different groups of patients, we used a Kaplan Meir curve and log-rank test. To identify mortality predictors, we used a cox regression analysis. We used SPSS-20 for all analyses.

**Results:**

A total of 326 patients died in the 12 years follow-up period contributing to 6.2% cumulative incidence and 21.7 deaths per 1000 person-year observations incidence rate. Eighty-nine percent of the total deaths were documented in the first two years follow up—an early-term ART follow up. Early HIV mortality rates among adults were 50% less in separated, divorced or widowed patients compared with never married patients, 1.6 times higher in patients with baseline CD4 count <200 cells/μL compared to baseline CD4 count ≥200 cells/μL, 1.5 times higher in patients with baseline WHO clinical stage 3 or 4 compared to baseline WHO clinical stage 1 or 2, 2.1 times higher in patients with immunologic failure compared with no immunologic failure, 60% less in patients with fair or poor compared with good adherence, 2.9 times higher in patients with bedridden functional status compared to working functional status, and 2.7 times higher with patients who had no history of HIV testing before diagnosis compared to those who had history of HIV testing. Most predictors of early mortality remained the same to the predictors of an overall HIV mortality. When discontinuation was assumed as an event, the predictors of an overall HIV mortality included age between 25–50 years, base line CD4 count, developing immunologic failure, bedridden functional status, and no history of HIV testing before diagnosis.

**Conclusions:**

The great majority of deaths were documented in the first two years of ART, and several predictors of early HIV mortality were also for the overall mortality when discontinuation was assumed as event or censored. Considering the above population, interventions to improve HIV program in the first two years of ART follow up should be improved.

## Introduction

Worldwide in 2015, 38.8 million people were living with human immunodeficiency virus (HIV), 2.5 million new HIV infections, and 1.2 million HIV/AIDS (acquired immunodeficiency syndrome) deaths were estimated, and sub-Saharan Africa (SSA) accounted for 76% of the global morbidity and 75% of the global mortality [1]. Ethiopia, one of the countries in SSA, had an estimate of 39,140 newly HIV-infected people, 768,040 people living with HIV, and 28,650 HIV/AIDS deaths in 2015 [1]. Ethiopia contributed 3% each to the global death and number of HIV-infected people in SSA respectively [2]. Antiretroviral therapy (ART) program in Ethiopia was commenced in 2003 in 12 hospitals at cost to the patients [3], and then since 2005, it was provided free of charge in 22 hospitals with the help of the international donors such as Global Fund, World Bank, and President's Emergency Plan for AIDS Relief (PEPFAR) [3, 4]. A total of 270,460 people were on ART in 913 health facilities in 2012–13 [3, 5] and the coverage rose to 339,043 adults and 22,955 children in 2014 [6].

The effectiveness of a country’s ART program depends on the HIV treatment cascade or HIV care continuum. HIV care continuum is a series of steps in which a person with HIV takes from initial diagnosis through their successful treatment with HIV medication [7, 8] that includes HIV diagnosis, assessment for ART eligibility, retention, and virological suppression. The success of The Joint United Nations Program on HIV and AIDS (UNAIDS) treatment targets—diagnosing 90% of people living with HIV, providing 90% of those diagnosed antiretroviral therapy (ART), and achieving viral suppression for 90% of patients receiving treatment—is affected by several factors [8–11]. Particularly, in order to meet the second and third 90s of the UNAIDS treatment targets, patient retention is a key program. However, attrition has been a routine impediment and remains monotonous in the thirty years of targeted HIV diagnosis and 20 years since ART rollout. Mortality from HIV, death of HIV-infected patients in the period of ART due to any cause, is one of the major contributors to attrition.

Recent global, regional and national estimates for mortality reported that 0.03 million deaths in high-income countries, 0.1 million deaths in middle-income countries, and 0.4 million deaths in low-income countries were recorded [1]. Similarly, several studies [12–19] that assessed the magnitude and predictors of mortality in Ethiopia are growing. Accordingly, the incidence of mortality has been reported between 2 and 25.9%, and the factors affecting for mortality of Ethiopian patients included but are not limited to male, primary level of education, single marital status, weight loss, bed-ridden functional status, low baseline cluster of differentiation 4 (CD4) count, advanced World Health Organization (WHO) clinical staging, tuberculosis (Tb)/HIV co-infection, severe anemia and substance abuse. In addition, the majority of the previous studies from Ethiopia reported high mortality is recorded in < 24 months since ART starting [13–15, 17, 20].

However, firstly, none of these studies assessed what predictors determined for the mortality in the early follow up periods. Secondly, none of the studies were also carried out in the southwestern part of the nation. Southwest Ethiopia has different cultural and socioeconomic characteristics, and according to the 2011 Ethiopian census, the regional state has the highest HIV prevalence (6.5%) in the nation than the other parts of the country (<2%) where the previous studies have been carried out. Therefore, the predictors that determine mortality may be different and need to be understood contextually to design interventions tailored to the individual regional states. Thirdly, all studies that assessed the predictors of mortality considered LTFU as censored, and none of them assumed the outcome of discontinued patients could be a death. Previous tracing studies from Ethiopia and Kenya reported that 40–86% of LTFU cases failed to re-engage to the care [21], and 50% of LTFU patients were found dead [22, 23]. Such failing to assume LTFU patients as deceased would lead to a biased estimate and spuriously low risk or odds ratio calculation. Fourthly, the great majority of the retrospective cohort studies from Ethiopia reported from a short-term durability of ART follow up periods or relatively low sample size.

The current study assessed the incidence and predictors of mortality in the early follow up periods using a 12-years data from the ART clinic at Jimma University Teaching Hospital (JUTH) in Southwest Ethiopia. The study also compared the predictors of early mortality with the cumulative mortality, and added another model assuming a worst-case scenario whereby all discontinued patients were assumed dead.

## Methods

### Study design, setting and participants

We used retrospective cohort study in ART clinic at JUTH in Southwest Ethiopia using patient records from June 2003 to March 2015. Details of the study setting has been described elsewhere [24–26]. All HIV-infected children and adults enrolled for ART care in JUTH were the target population. The details of the treatment protocol for Ethiopia is described elsewhere [27].

### Data source and procedures

**Fig 1:**
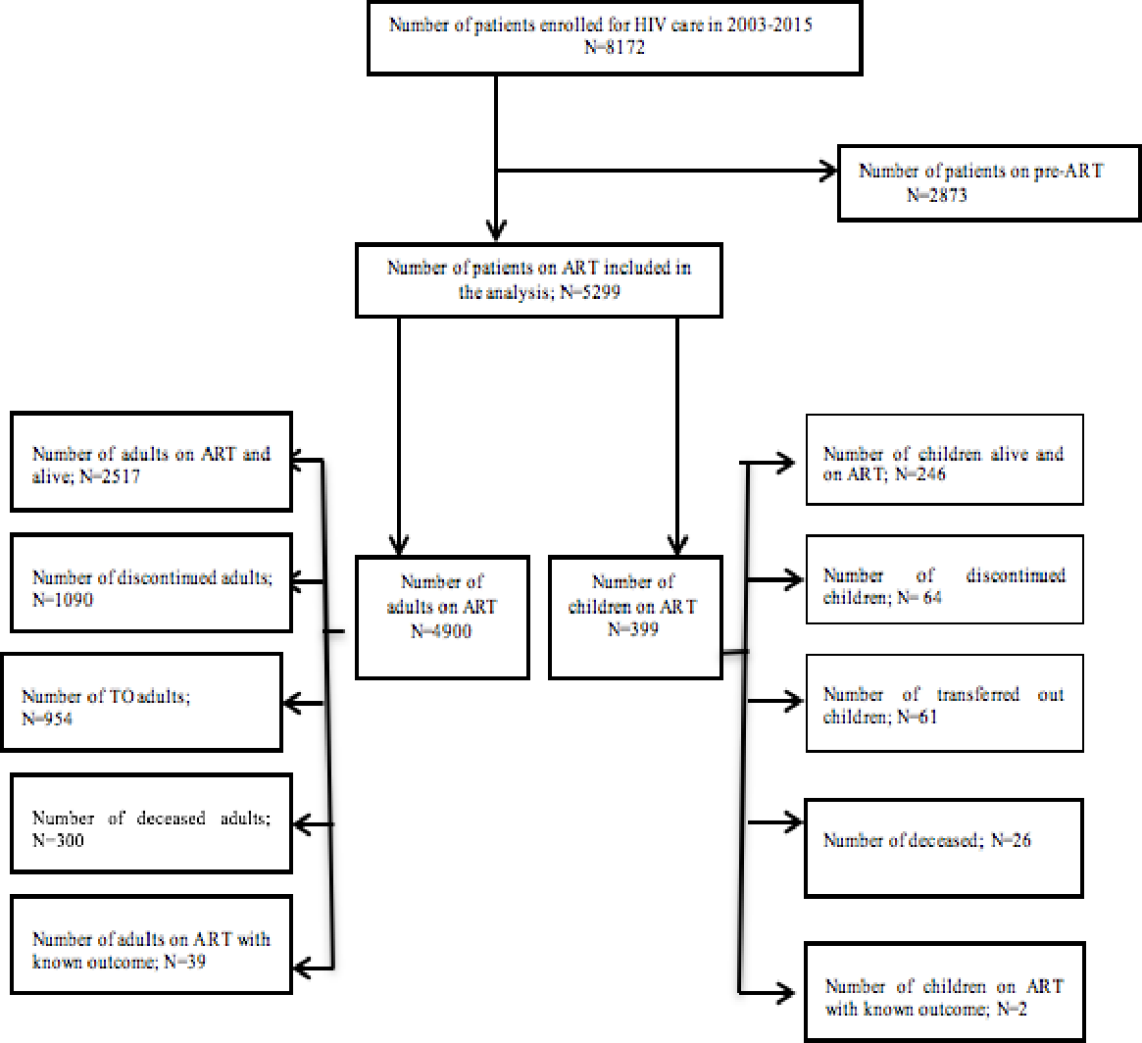
Schematic presentation of data extraction of HIV infected patients on ART in 2003–15 in Jimma University Teaching Hospital, Southwest Ethiopia—This figure shows the data extraction flowchart.

We extracted the data from JUTH electronic medical records (EMR) system designed since 2007. Clinicians record clinical and non-clinical information of patients on paper form, and then data clerks entered into the EMR system. Two data clerks undertake the data entry process to warrant completeness. The International Center for AIDS Care and Support (ICAP) at Colombia University has been delivering technical assistance on the electronic patient level data management, and carrying out check up of data completeness. This ensures the accuracy and reliability of the EMR data. If outcome status of a patient were not recorded or transferred out, records would be excluded from the analysis. Fig 1 demonstrates the schematic presentation of data extraction HIV infected children and adult enrolled in the period 2003–2015 in Jimma University Teaching Hospital, Southwest Ethiopia.

### Study variables and measurements

The response variables were the survival time in months and events related to HIV. ART patients were followed until the date of the event, discontinuation or the end of the study. We defined mortality as the death of people on ART in the reporting period due to any cause [22]. Patients who are alive and on ART until March 2015, and discontinued from ART—LTFU, defaulting and stopping medication—were considered as a censored i.e. they were assumed to be alive for the time period they had been under follow up. ART outcomes—mortality, discontinuation or alive and on ART—were the outcomes that were recorded in the final date of the data collection. ART duration was dichotomized as early and short. Short-term follow up period was defined if the ART follow-up period was below 24 months [22]. The independent variables included age, sex, marital status, educational status, religion, ART adherence, Cotrimoxazole adherence, late presentation for HIV care (LP), clinical failure, immunological failure, treatment failure, Tb/HIV co-infection, baseline functional status, history of HIV testing and ART shift. History of HIV testing refers to testing (one or more times) for HIV before HIV diagnosis. ART shift is switching of first line to second line ART regimen. Attrition refers to a condition where HIV-infected patients fail to retain in care i.e. mortality, lost to follow up from ART, defaulting from ART or stopping ART. Table 1 reports the operational definitions for variables related to attrition (mortality and discontinuation). Table 2 reports the measurements of LP, adherence, clinical, immunological and treatment failures.

**Table 1:** Measurements for variable related to death and ART attrition (mortality and discontinuation).

Variable	Definition	Numerator	Denominator
Cumulative incidence	The number of deaths among patients enrolled on ART during the follow-up	Number of deaths during the entire ART follow-up period (2003–15)	Number of patients during the entire follow up period (2003–15)
Incidence rate	Number of deaths among patients enrolled on ART in a person-time observations	Number of deaths during the entire follow up period	Time each person was observed, totaled for all persons (total person-years observations)
Annual death rate	The death rate in a specific calendar year among patients enrolled on ART during that calendar year	Number of deaths in a specific calendar year	Number of patients died plus alive and on ART plus discontinued plus transferred out during the specific calendar year
Calendar year	The year which the death rate is calculated	Not applicable (NA)	NA
LTFU	If patients had been on ART treatment and had missed at least three clinical appointments but had not yet been classified as “dead” or “transferred out (transferred)”.	NA	NA
Defaulting	If patients had been on ART treatment and had missed less than three clinical appointments but had not yet been classified as “dead” or “TO”.	NA	NA
Stopping medication	If patients had stopped treatment due to any reason while they have remained in care.	NA	NA
Transfer out	Transferred is the official transferring of the patient to another ART clinic within or outside a catchment area.	NA	NA

ART: antiretroviral therapy; LTFU; lost to follow up; NA: not applicable

**Table 2:** Measurements for late presentation for HIV care, ART or cotrimoxazole adherence, and immunological, clinical & treatment failures.

Adults	**Late presentation for HIV care**[28–30] ^a^			
	**Enrolled in 2003–11**		**Enrolled in 2012–15**	
	CD4 lymphocyte count of <200 cells/μL irrespective of WHO clinical stage at the time of first presentation to the HIV care		CD4 lymphocyte count of <350 cells/μL irrespective of WHO clinical stage at the time of first presentation to the HIV care	
	WHO clinical stage 3 or 4 irrespective of CD4 count at the time of first presentation to the HIV care ^b^		WHO clinical stage 3 or 4 irrespective of CD4 count at the time of first presentation to the HIV care ^b^	
Children ^c^	**Late presentation for HIV care**[31]			
		**Moderate immuno-suppression if CD4 count between**	**Severe immunosuppression if CD4 count between**	
	0–12 months	750–1500 cells/μL	<750 cells/μL	
	1–5 years	500–1000 cells/μL	<500 cells/μL	
	≥ 6 years	200–500 cells/μL (enrolled in 2003–2011)	<200 cells/μL (enrolled in 2003–11)	
	≥ 6 years	350–500 cells/μL (enrolled in 2012–2015)	<350 cells/μL (enrolled in 2012–2015)	
Children and adults	**Adherence status to ART or cotrimoxazole**[32]^d^			
	**Status**	**Percentage of prescribed ART intake**	**Number of missing doses out of 30**	**Number of missing doses out of 60**
	Good	≥ 95%	<3	<4
	Fair	85–95%	3–5	4–9
	Poor	< 85	≥6	≥9
	**Clinical, immunological and treatment failures for children and adults** [33]			
**Clinical failure**			**Immunological failure**	**Treatment failure**
Children	New clinical condition indicating severe immunodeficiency (WHO clinical stage 3 and 4 with the exception of Tb) after 6 months on ART		Persistent CD4 counts <200 cells/mm^3^ for children age<5 years, or <100 cells/mm^3^ for children age≥ 5 years	Having both clinical and immunological failures
Adults	New clinical condition indicating severe immunodeficiency (WHO clinical stage 4) after 6 months on ART		If CD4 count of the HIV-infected adults falls to the baseline (or below) or persistent CD4 levels below 100 cells/mm3 after six months of ART treatment	

ART: antiretroviral therapy; CD4: cluster for differentiation 4; WHO: World Health Organization; Tb: Tuberculosis; PCP: pneumocystis carinii (juvenii) pneumonia

^a^The definition for LP among Tb/HIV co-infected population was only based on the CD4 criteria[25].

^b^ **WHO clinical Stage 3** was defined if one of the following is present in an HIV diagnosed patient: weight loss of >10% body weight, chronic diarrhoea for >1 month, fever for >1 month, oral candidiasis, oral hairy leukoplakia, or pulmonary Tb within the previous year, or severe bacterial infections; **WHO clinical Stage 4** was defined if one of the following is present in an HIV diagnosed patient:HIV wasting syndrome, PCP, toxoplasmosis of the brain, cryptosporidiosis or isosporiasis with diarrhea for >1 month, cytomegalovirus disease of an organ other than liver, spleen or lymph node, herpes simplex virus infection, progressive multifocal leukoencephalopathy, candidiasis, extra-pulmonary Tb, lymphoma, kaposi’s sarcoma, HIV encephalopathy

^c^LP is also defined if WHO clinical stage 3 or 4 at first visit to the ART clinics

^d^ Clinicians/pharmacists ask patients and check the pill container to collect the number of missing doses or days.

### Statistical analyses

We edited and cleaned up the data, to summarize the characteristics of cohort, we used descriptive statistics that included median and range values for continuous data; percentage, frequency tables, and graphs for categorical data. The calculation for cumulative incidence (CI) and incidence rate (IR) of mortality is presented in Table 1. For children, the event (death) was occurred only in 26 participants, and this small event occurrence does not allow us to conduct further inferential statistics—the cox regression analysis. For adults, we used Kaplan Meir curve to estimate survival time and compare the time to event among the different groups of patients. The log-rank test was used to check any significant differences in survival among different levels of the categorical variables measured in the study. We calculated the estimated survival time in months using the time between date of treatment initiation and date of death or censoring.

We applied bivariate cox regression analysis to see the existence of crude association and select candidate variables (with *P* value below 0.25) to multiple cox regression. We carried out a multiple cox regression analysis to identify independent predictors of mortality using a stepwise variable selection. We assessed the assumption for proportional hazard graphically. We checked the goodness of fit of the final model using Hosmer and Lemeshow test and was found fit. We also checked the collinearity diagnosis between selected independent variables. *P*-value of <5% was considered significant in the final model. Three models were constructed. Model I shows the predictors of early mortality among HIV-infected patients attending short-term (<24 months) ART follow-ups. Model II—real case assumption—shows the predictors of an overall mortality (cumulative) among HIV-infected patients attending ART assuming discontinuation as censored. Model III—worst case assumption or intention-to-treat analysis—shows the predictors of an overall mortality (cumulative) among HIV-infected patients attending ART assuming discontinuation as event(death) in addition to the real event. We used Statistical Package for the Social Sciences (SPSS) version 22.0 for all data analyses.

### Ethical approval

The ethical clearance was obtained from the Social and Behavioral Research Ethics Committee (SBREC) at Flinders University (Project number: 7086) and Institutional Review Board (IRB) of College of Health Sciences at Jimma University (Ref No: RPGC/386/2016). A de-identified data was extracted from the database, and its access permission was obtained from JUTH board.

## Results

The study included 8,172 ART patients enrolled from 21 June 2003 to 15 March 2015, with a median follow up times of 49 months. Of total, 5,299 (64.8%) patients were on ART of whom 4,900 (92.5%) were adults and 399 (7.5%) were children. Table 3 presents the clinical and non-clinical characteristics of HIV-infected adults and children on ART. Of the children, the majority (58.1%) were age 5-<15 years, half (52.4%) were males, and three-quarter (73.9%) were Christians. Four out of five children (79.4%) had moderate or severe immunosuppression, and half (50.5%) of the children had baseline WHO clinical stage 3 or 4. The prevalence of Tb/HIV co-infection among children was 28.6%.

**Table 3:** Clinical & non-clinical characteristics of HIV infected people enrolled in ART care in Southwest Ethiopia from 2003–15.

Variable		Children (N = 399)	Adult (N = 4900)
		N (%)	N (%)
Age in years	<1	21 (5.3)	
	1-<5	146 (36.6)	
	5-<15	232 (58.1)	
	15-<25		711 (14.5)
	25-<50		3937 (80.3)
	50+		252 (5.2)
	Median (range) age in years	6 (<1–14)	30 (15–81)
Sex	Male	209 (52.4)	1971 (40.2)
	Female	190 (47.6)	2929 (59.8)
Marital status^b^	Never married		897 (20.9)
	Married		2094 (48.7)
	Separated/divorced/widowed		1311 (30.5)
Education^b^	No education		945 (21.9)
	Primary		1687 (39.1)
	Secondary and above		1685 (39)
Religion^b^	Muslim	47 (26.1)	1402 (32.6)
	Christian^a^	133 (73.9)	2893 (67.4)
Baseline WHO classification ^b^	1 or 2	108 (49.5)	1355 (45.7)
	3 or 4	110 (50.5)	1608 (54.3)
Baseline CD4 count category ^b^	No damage	72 (20.6)	
	Moderate or severe damage	277 (79.4)	
	Median (range) CD4 count	282 (0–2250)	
Baseline CD4 count (cells/mm3) ^b^	<200		3275 (73.6)
	≥ 200		1174 (26.4)
	Median (range)		156 (0–1313)
History of Tb/HIV co-infection ^b^	No	285 (71.4)	3533 (72.1)
	Yes	114 (28.6)	1367 (27.9)
ART adherence ^b^	Good	319 (79.9)	4064 (82.9)
	Fair or poor	80 (20.1)	836 (17.1)
Cotrimoxazole adherence ^b^	Good	315 (78.9)	4119 (94.4)
	Fair or poor	84 (21.1)	762 (15.6)
History of HIV testing ^b^	Yes	399 (100)	2860 (58.4)
	No	0 (0)	2040 (41.6)
ART shift ^b^	No	214 (97.7)	3190 (99.1)
	Yes	5 (2.3)	29 (0.9)
Baseline functional status ^b^	Appropriate	170 (42.6)	
	Delay or regression	229 (57.4)	
Baseline functional status ^b^	Work or Ambulatory		3064 (68.1)
	Bedridden		1437 (31.9)
HIV care presentation	Early	162 (43)	894 (33.3)
	Late	215 (57)	1788 (66.7)
Clinical failure ^b^	No	165 (77.1)	2261 (80.5)
	Yes	49 (22.9)	546 (19.5)
Immunologic failure ^b^	No	295 (84.8)	3164 (80.3)
	Yes	53 (15.2)	775 (19.7)
Treatment failure ^b^	No	126 (31.6)	1493 (65.7)
	Yes	61 (15.3)	780 (34.3)
Duration of ART	Short (<24 months)	143 (7.8)	1697 (92.2)
	Long (> = 24 months)	193 (8)	2210 (92)

^a^Orthodox, Catholic, Protestant

^b^ only valid percentage is calculated; ART: antiretroviral therapy; CD4: cluster for differentiation 4; WHO: World Health Organization; Tb: Tuberculosis

The median time on ART was 40 months where as the estimated survival time was 104.2 (99.8–108.5) months. Of the adults, 59.8% were females, 48.7% were married and 39% had primary school education. Three quarters (73.6%) of HIV-infected adults had baseline CD4 count below 200 cells/mm^3^, and 54.3% had WHO clinical stage 3 or 4. Tb/HIV co-infection was diagnosed in about a quarter of adults (27.9%). In addition, 29(0.9%) HIV-infected adults were switched to second line ART drugs.

### Cumulative incidence (CI) and incidence rate (IR) of mortality in HIV-infected patients

Of the 5299 ART patients, 2763 (52.5%) patients were alive and on ART, 1154 (21.9%) patients discontinued from the treatment, and 1015 (19.3%) patients transferred to other sites by the end of march 2015(Table 4). The remaining 326 (6.2%) patients died contributing to a CI of 6.5% (26/399) in children and 6.1% (300/4900) in adults. Of the total deaths, 220 deaths (67.5%) occurred in the first six months of ART follow up, 37 (11.3%) occurred in between 6-<12 months, 32 (9.8%) occurred in between 12-<24 months and the remaining 37 (11.3%) deaths occurred ≥24 months.

**Table 4:** Annual number of patients enrolled in ART care and their outcomes.

Year	New enrolment (a)	Outcome final, n (%)				
		Death (b)	Discontinuation (c)	Transfer out (d)	Alive and on ART (e)	Total in Cohort (f)
2003	8	0 (0)	1 (12.5)	0 (0)	7 (87.5)	8
2004	62	1 (1.4)	7 (10.1)	1 (1.4)	60 (87.0)	69
2005	484	28 (5.1)	51 (9.4)	9 (1.7)	456 (83.8)	544
2006	973	66 (4.6)	90 (6.3)	71 (5.0)	1202 (84.1)	1429
2007	622	53 (2.9)	155 (8.5)	137 (7.5)	1479 (81.1)	1824
2008	555	45 (2.2)	112 (5.5)	97 (4.8)	1780 (87.5)	2034
2009	566	42 (1.8)	54 (2.3)	109 (4.6)	2141 (91.3)	2346
2010	481	23 (0.9)	152 (5.8)	81 (3.1)	2366 (90.2)	2622
2011	461	29 (1.0)	93 (3.3)	112 (4.0)	2593 (91.7)	2827
2012	383	11 (0.4)	101 (3.4)	103 (3.5)	2761 (92.8)	2976
2013	324	17 (0.6)	117 (3.8)	107 (3.5)	2844 (92.2)	3085
2014	320	9 (0.3)	179 (5.7)	158 (5.0)	2818 (89.1)	3164
2015	60	2 (0.1)	42 (1.5)	30 (1.0)	2763 (97.4)	2878
Overall		326 (6.2)	1154 (21.9)	1015 (19.3)	2763 (52.5)	5299

e = f-b-c-d; where f = e (previous year) + a (current year); The annual percentages for death, discontinuation, transfer out, and alive and on ART is calculated by dividing the number of patients on ART with respective outcomes in a calendar year to the number of patients on ART in the cohort in that calendar year.

**Fig 2:**
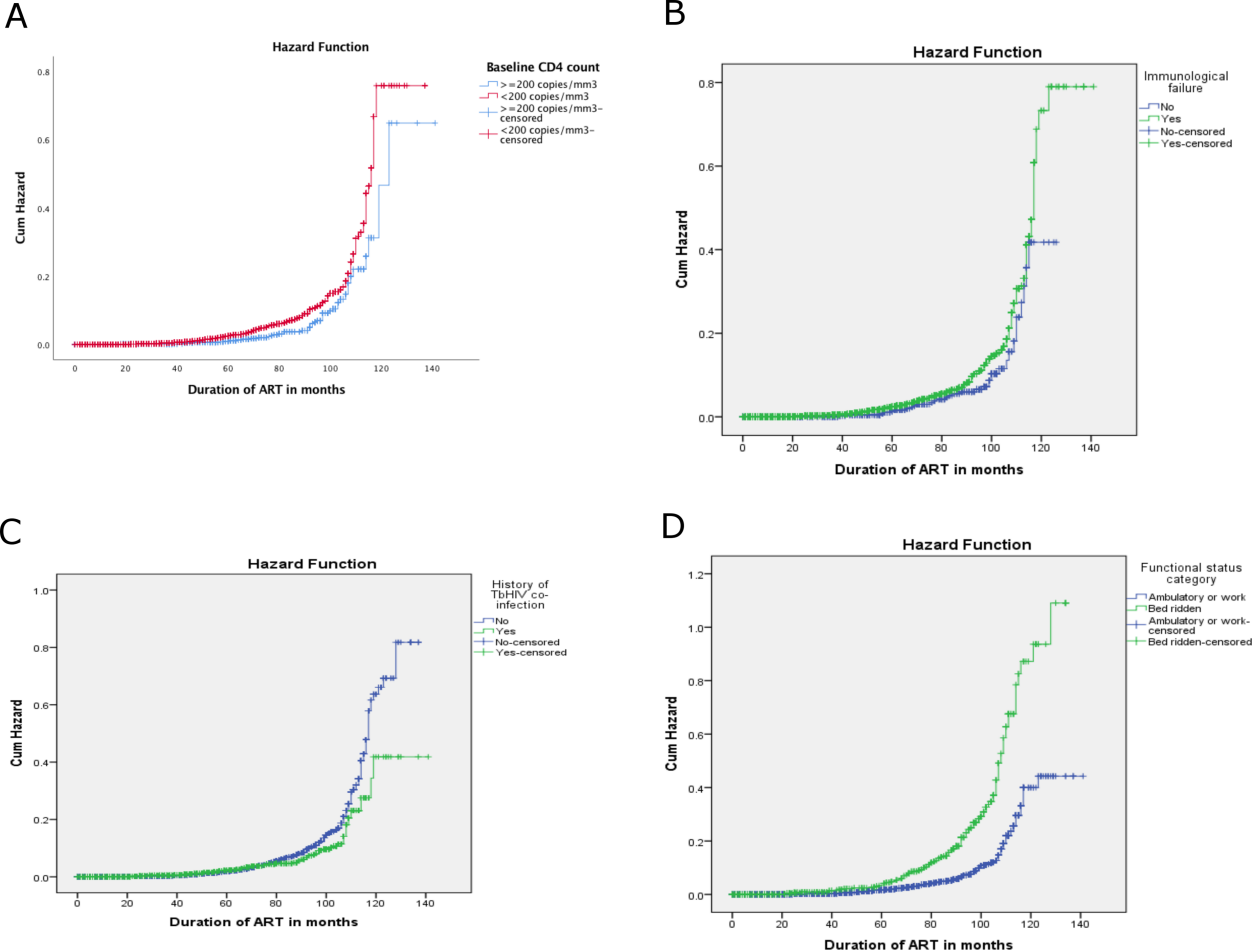
**(a-d) Kaplan-Meier plot of hazard function stratified according to baseline CD4 count, immunologic failure, Tb/HIV co-infection and functional status among a cohort of ART clients respectively; JUTH hospital, Southwest Ethiopia; 2016**—This figure presents the association between baseline CD4 count **immunologic failure, Tb/HIV co-infection and functional status,** and HIV mortality.

The total follow-up period encompassed 15, 051 person-years observations, and an estimated survival time of 121.9 (95%CI: 120.3–123.5) months. The overall IR was 21.7 deaths (22.2 deaths for children and 21.6 deaths for adults) per 1000 person-years observations. The magnitude of mortality had peaked in 2006 but reduced significantly and remained low in subsequent years of follow-up (Table 4). Fig 2A–2D respectively show mortality status of study participants by baseline CD4 count, immunologic failure, history of Tb/HIV co-infection and functional status using Kaplan-Meier graphs. Accordingly, the hazard distribution of ART clients for sex, baseline CD4 count and history of Tb/HIV co-infection was statistically significant.

### Predictors for mortality among adult HIV patients on ART

Table 5 demonstrates the findings from three models of the cox regression analysis. Model I presents results of the bivariate and multivariable cox regression analysis of predictors for early mortality among adult HIV patients attending short-term ART follow-ups. Predictors of early mortality included being separated/divorced/ widowed, having a baseline CD4 count below 200 cells/μL, baseline WHO clinical staging, developing immunologic failure, fair or poor ART adherence, bedridden functional status and no history of HIV testing. HIV patients who were separated, divorced, or widowed were 50% less likely (AHR = 0.5, 95%CI: 0.3–0.8) to die compared to those who never married. Compared to patients with baseline CD4 count 200 cells/μL and above, the hazard of death was higher (AHR = 1.6, 95%CI: 1.05–2.5) among patients with baseline CD4 count <200 cells/μL. In addition, compared to patients with baseline WHO clinical stage 3 or 4, the hazard of death was higher (AHR = 1.5, 95%CI: 1.05–2.5) among patients with baseline WHO stage 1 or 2. Patients who developed immunologic failure were two times (AHR = 2.1, 95%CI: 1.4–3.01) at risk to die than those who did not develop immunologic failure. The hazard of death was 40% less among patients with poor or fair ART adherence compared to those with good adherence. Patients with bedridden functional status were more likely (AOR = 2.9, 95%CI: 2.02–4.07) to die than their comparator. Patients with no history of HIV testing before diagnosis had also a higher risk of mortality (AHR = 2.7, 95%CI: 1.9–3.8) than those who had history of HIV testing.

**Table 5:** Factors related to HIV-associated mortality among HIV-infected adults in 2003–2015, JUTH, Southwest Ethiopia.

Variable		Status (Short-term follow up)		Model I: <24 months of follow-up (Short-term follow up)		Model II: Cumulative (0–140 months of follow-up) (Real case assumption)		Model III: Cumulative (0–140 months of follow-up) (Worst case assumption)	
		Censored, n(%)	Event, n(%)	CHR (95%CI)	AHR (95%CI)	CHR (95%CI)	AHR (95%CI)	**CHR (95%CI)**	**AHR (95%CI)**
**Age**	15–25 years)	554 (95.2)	28 (4.8)	Reference	Reference	Reference	Reference	Reference	Reference
	25–50 years	2859 (92)	249 (8)	0.9 (0.7–1.00)	0.8 (0.6–1.2)	1.3 (0.9–1.9)	1.3 (0.9–1.9)	0.8 (0.7–0.98) ^a^	0.8 (0.6–0.9) ^a^
	50+ years	194 (89.4)	23 (10.6)	1.2 (0.9–1.6)	1.1 (0.7–1.8)	2.5 (1.4–4.3) ^a^	1.9 (0.4–3.3)	1.2 (0.9–1.6)	0.8 (0.4–1.5)
**Sex**	Male	1385 (91.2)	134 (8.8)	Reference	-------	Reference	-------	Reference	-------
	Female	2222 (93)	166 (7)	1.02 (0.9–1.1)	-------	1.06 (0.84–1.3)	-------	1.02 (0.9–1.1)	-------
**Marital status**	Never married	686 (90.7)	70 (9.3)	Reference	Reference	Reference	Reference	Reference	Reference
	Married	1503 (91)	149 (9)	1.4 (1.2–1.6) ^a^	0.8 (0.5–1.1)	0.99 (0.7–1.3)	0.7 (0.5–1.05)	1.4 (1.2–1.6) ^a^	1.3 (0.9–1.7)
	Other ^b^	896 (93.6)	6.4	1.2 (1.06–1.5) ^a^	0.5 (0.3–0.8) ^a^	0.7 (0.5–1.02)	0.5 (0.2–0.9) ^a^	1.2 (1.06–1.5) ^a^	1.09 (0.9–1.5)
**Educational status**	No education	679 (94.4)	40 (5.6)	Reference	-------	Reference	-------	Reference	Reference
	Primary	1227 (91.8)	109 (8.2)	1.00 (0.8–1.1)	-------	1.6 (0.97–2.2)	-------	0.9(0.8–1.2)	1.00 (0.7–1.4)
	Secondary & above	1197 (89.9)	135 (10.1)	1.2 (1.01–1.4) ^a^	-------	1.9 (0.8–2.7)	-------	1.2 (1.01–1.4) ^a^	1.3 (0.9–1.8)
**Religion**	Muslim	1026 (92.3)	85 (7.7)	Reference	-------	Reference	-------	Reference	-------
	Christian ^c^	2060 (91.3)	197 (8.7)	1.01 (0.9–1.1)	-------	1.2 (0.9–1.5)	-------	1.009 (0.9–1.1)	-------
**Baseline WHO status**	1 or 2	982 (91.5)	91 (8.5)	Reference	Reference	Reference	Reference	Reference	Reference
	3 or 4	1123 (90.6)	116 (9.4)	1.07 (0.9–1.2)	1.5 (1.05–2.01) ^a^	1.0 (-1.4)	1.1 (0.6–1.5)	1.07 (0.9–1.2)	1.1 (0.9–1.2)
**Baseline CD4 count**	> = 200 cells/μL	907 (95.3)	45 (4.7)	Reference	Reference	Reference	Reference	Reference	Reference
	<200 cells/μL	2388 (91.6)	218 (8.4)	1.3 (1.1–1.5) ^a^	1.6 (1.05–2.5) ^a^	1.6 (1.1–2.1) ^a^	2.01 (1.5–3.5) ^a^	1.3 (1.1–1.5) ^a^	1.1 (1.07–1.3)
**Clinical failure**	No	1652 (91.6)	151 (8.4)	Reference	-------	Reference	-------	-------	-------
	Yes	388 (88.6)	50 (11.4)	0.8 (0.7–1.0)	-------	1.03 (0.7–1.4)	-------	-------	-------
**Immunologic failure**	No	617 (94.9)	33 (5.1)	Reference	Reference	Reference	Reference	Reference	Reference
	Yes	2534 (92)	220 (8)	1.5 (1.3–1.8) ^a^	2.1 (1.4–3.01) ^a^	1.4 (0.9–2.02)	1.2 (0.5–2.4)	1.5 (1.3–1.8) ^a^	1.4 (1.09–1.8) ^a^
**HIV care presentation**	Early	643 (90.8)	65 (9.2)	Reference	-------	Reference	-------	Reference	
	Late	1274 (91.2)	119 (8.5)	1.00 (0.8–1.1)	-------	1.01 (0.7–1.2)	-------	1.00 (0.8–1.1)	
**ART duration**	Short	-------	-------	-------	-------	Reference	Reference	-------	-------
	Long	-------	-------	-------	-------	0.07 (0.05–0.11) ^a^	0.08 (0.05–0.1) ^a^	-------	-------
**Tb/HIV co-infection**	No	2546 (91.5)	238 (8.5)	Reference	Reference	Reference	Reference	Reference	Reference
	Yes	1061 (94.5)	62 (5.5)	0.85 (0.75–0.97) ^a^	0.7 (0.6–0.9)	0.7 (0.5–0.9) ^a^	0.9 (0.6–1.3)	0.85 (0.76–0.97) ^a^	1.1 (0.9–1.4)
**ART adherence**	Good	2938 (91.8)	264 (8.2)	Reference	Reference	Reference	-------	Reference	Reference
	Fair or poor	667 (94.9)	36 (5.1)	0.7 (0.6–0.8) ^a^	0.4 (0.2–0.7) ^a^	0.6 (0.5–0.8) ^a^	-------	0.7 (0.6–0.8) ^a^	0.9 (0.7–1.1.3)
**Cotrimoxazole adherence**	Good	2983 (91.7)	269 (8.3)	Reference	Reference	Reference	-------	Reference	Reference
	Fair or poor	609 (95.3)	30 (4.7)	0.8 (0.7–0.9) ^a^	0.7 (0.4–1.09)	0.6 (0.4–0.9) ^a^	-------	0.8 (0.7–0.9) ^a^	0.8 (0.6–1.3)
**Functional status**	Working/Ambulatory	3103 (94.8)	171 (5.2)	Reference	Reference	Reference	Reference	Reference	Reference
	Bedridden	442 (81.1)	103 (18.9)	2.5 (2.3–2.8) ^a^	2.9 (2.02–4.07) ^a^	2.8 (2.1–3.6) ^a^	2.2 (1.4–3.9) ^a^	2.5 (2.3–2.9) ^a^	3.00 (2.3–4.0) ^a^
**History of HIV testing**	Yes	2079 (93.2)	152 (6.8)	Reference	Reference	Reference	Reference	Reference	Reference
	No	1528 (91.2)	148 (8.8)	1.9 (1.7–2.1) ^a^	2.7 (1.9–3.7) ^a^	2.9 (2.3–3.6) ^a^	2.7 (1.9–3.8) ^a^	1.9 (1.7–2.1) ^a^	1.9 (1.6–2.4) ^a^
**ART shift**	Yes	2302 (91)	228 (9)	-------	-------	-------	-------	-------	-------
	No	29 (100)	0 (0)	-------	-------	-------	-------	-------	-------

^a^ statistically significant at p-value ≤0.05

^b^ Separated/divorced/widowed

^c^ Orthodox, Protestant or Catholic

Model II—real case assumption—reports results of the bivariate and multiple cox regression analysis of predictors for overall mortality (cumulative) among HIV patients attending ART assuming discontinuation and alive as censored. Predictors of mortality included being separated/widowed/divorced, having baseline CD4<200cells/μL, short ART duration, bedridden functional status and no history of HIV testing. Females had 40% lesser probability (AHR = 0.5, 95%CI: 0.3–0.8) to die than males. HIV-infected patients who were separated, divorced or widowed were less likely (AHR = 0.5, 95%CI: 0.2–0.9) to die than those who never married. Patients with baseline CD4 count <200 cells/μL had an elevated risk of death (AOR = 2.01, 95%CI: 1.5–3.5) than those with baseline CD4 count 200 cells/μL and above. HIV patients with longer ART duration has less likely (AHR = 0.08, 95%CI: 0.05–0.1) to die than those who were on short ART duration. Having bedridden functional status (AHR = 2.2, 95%CI: 1.4–3.9) and no history of HIV testing (AHR = 2.7, 95%CI: 1.9–3.8) were also another risk factors for the overall mortality.

Model III—worst case assumption or intention-to-treat approach—presents results of the bivariate and multiple cox regression analysis of predictors for overall mortality among HIV-infected patients attending ART assuming discontinuation as event. In addition to baseline CD4 count, functional status and history of HIV testing—predictors of mortality in the real case assumption—, age and immunological failure had statistically significant association when discontinuation is assumed as an event.

## Discussion

In this study, the cumulative incidence mortality for HIV-infected patients on ART was found to be 6%, which is lower than was reported in studies carried out in another parts of Ethiopia such as Tigray [15], Southern Nations, Nationalities and Peoples Region (SNNPR)[19] and Amhara [34], where the CI were 9%, 10% and 41% respectively. Despite that, participants of the current study setting are diversified (i.e. from high HIV prevalence rate settings, low HIV prevalence rate settings, and refugee camps), the magnitude of death was not higher than from the other settings. This may be attributed to several reasons. First, the magnitude of death may be attributed to the level of late HIV care presentation. For example, 65% of the participants in the present study were late presenters as compared to 69% in Tigray [35]. Second, the magnitude of death may be attributed to the level of adherence to ART. For example, 20% of the participants in the present study were non-adherent as compared to 26% in the SNNPR [36].Third, the magnitude of death may be attributed to the magnitude of Tb/HIV co-infection. For example, 28% of the participants in the present study had Tb/HIV co-infection as compared to 44% in Amhara [37].

The majority of deaths happened during the first 6 months after treatment initiation, but reduced substantially in the first year of treatment, and remained low in subsequent years of follow-up as reported elsewhere [19, 22, 38–40]. This has an implication with the criteria for ART initiation. The treatment protocol for Ethiopia is implemented using WHO ART treatment guideline [41] and National Guidelines for Comprehensive HIV Prevention, Care and Treatment: Federal Democratic Republic of Ethiopia, Ministry of Health [42]. These protocols consider baseline CD4 count and/or WHO clinical staging. According to these protocols, patients were used to wait until their CD4 count and/or WHO clinical staging dropped down to the criteria. Recently, since the end of 2016, the initiation of test and treat strategy in the country will have an impact on reducing early HIV mortality. This program should be strengthened throughout the nation. The HIV mortality had peaked in 2005–07, and this could possibly be: i) the free introduction of ART in 2005–6 in Ethiopia was without intensive preparation [22]; ii) high rate of late HIV diagnosis (70–74%) was recorded in the period in the current study; iii) there was poor awareness about the modern medicine, and to the contrary, traditional medicine was more well-known and accessible in Ethiopia[43]; iv) negative belief about ART treatment could be another reason [44]; and v) there was a scarcity of ART supply and the referral linkage was very poor since the system was at an early stage. Since 2008, the HIV mortality has markedly declined as reported by the previous studies [45–47]. Since the scale up of ART in 2005, the overall mortality rate decreased by 40%, 85% and 100%, respectively, in 2007, 2011 and 2014. The Government of Ethiopia that has made a significant improvement in health infrastructure—health-care institutions, laboratories, and capacity building of health professionals—has contributed to the significant reduction of HIV mortality [3, 5]. The remarkable reduction of death could also be attributed to the introduction of effective combination of ART [48–50],early initiation of ART due to lowering the CD4 based ART initiation criteria [51], and the expansion of ART programs to primary health care facilities [52].

Findings of the current study, as consistent as other findings, revealed that predictors of early mortality included no history of HIV testing [53], low baseline CD4 count[19, 54, 55], advanced WHO clinical stage [22, 55], immunologic failure[56] and bedridden functional status [14, 19, 22]. Such patients with low CD4 count, advanced WHO clinical stage, immunologic failure and bedridden functional status are vulnerable to advanced stage of disease and subsequently death [22]. Additionally, patients who had no history of HIV testing could be diagnosed late, and rapidly progress to advanced stage of the HIV/AIDS[53]. This particularly calls for earlier HIV diagnosis and timely initiation of ART, and generally cues consolidation of the HIV care continuum to diminish early HIV-related mortality[19]. In the present study, it is very surprising that patients with fair or good adherence has less probability of dying than those HIV patients with good adherence, and this needs further study. Despite the development of significant immunological or clinical failure, only 29 (0.9%) patients were switched to second line ART drugs, as explained in a previous study[54] that reported 6 (0.2%) patients were switched to second line ART drugs.

The study should be interpreted in light of the following strengths and limitations. The study included all age groups, very large sample and long follow up times (since the commencement of ART history), and these increase the power to detect differences in mortality by the study variables. In addition, the study participants involved in the current study area were from different socio-demographic characteristics. There are a number of HIV-infected people from Gambella, a regional state where the highest prevalence of HIV (6.5%) was recorded. On the other hand, majority of people were from Oromiya regional state, where the prevalence of HIV is similar (1.2–2%) to the other regional states of the nation. A considerable number of people were also attending the HIV clinic from a refugee camp located near Jimma.

However, the following limitations should be taken in to consideration: (1) being a single reference center may not reflect the situation of a whole country; (2) the CI of mortality might slightly be affected, as outcome status of 32 patients (2 children and 30 adults) was not recorded; (3) there might be a misclassification bias of deaths among discontinued patients; (4) the intention-to-treat analysis may underestimate survival functions since all discontinued patients were assumed dead; (5) The data for analysis used date back to 2015, and this did not include data after the *‘test and treat’* strategy has been initiated; and (6) the association of some important variables such HIV-related stigma that has significant impact in the cascade of HIV care [57] was not assessed due to retrospective nature of the study design.

## Conclusions

The magnitude of mortality was considerable (21.7 deaths per 1000 person-years), and majority of deaths (89%) occurred within 24 months of ART follow-up; however, the annual rate of mortality has been decreasing significantly. Thus, to retain patients long with a favourable quality of life, a thoughtful consideration should be given to the early HIV care services targeting to the above-mentioned predictors. Predictors of early mortality were also predictors of the overall mortality even in the intention-to-treat analysis. This suggests that applying interventions focusing on these predictors will reduce not only the death of patients attending ART care but also those who had discontinued.

## Abbreviations

AIDS: acquired immunodeficiency syndrome
ART: antiretroviral therapy
HIV: human immunodeficiency virus
HCC: HIV care continuum
JUTH: Jimma University Teaching Hospital
LTFU: loss to follow up
MIs: multiple imputations
SSA: Sub-Saharan Africa
Tb: tuberculosis
UNAIDS: The Joint United Nations Program on HIV and AIDS
WHO: World Health Organization
